# Supplementary material for: Optimizing hyperparameters of YOLOv10 for arson detection using advanced optimization algorithms
Source: PLoS One. 2025 Oct 7;20(10):e0333374. doi: 10.1371/journal.pone.0333374 (PMC12503287; doi:10.1371/journal.pone.0333374)
Supplement: Attachment — Submitted filename: Response to Reviewers.docx [file pone.0333374.s002.docx]

***Response to Reviewers***

**Manuscript Title:** Optimizing Hyperparameters of YOLOv10 for Arson Detection Using Advanced Optimization Algorithms

**Manuscript Number:** PONE-D- 25-07942

We would like to express our sincere appreciation to the editor and the reviewers for their insightful comments and valuable suggestions, which have greatly contributed to enhancing the clarity and overall quality of our manuscript. We have carefully addressed all concerns and implemented the recommended changes accordingly. The following sections present our detailed responses and highlight the modifications made based on the reviewers' feedback. We are grateful for your constructive input and the opportunity to improve our work through this review process.

**Academic Editor**

We acknowledge your comments and have taken them into sincere consideration. Below is our response to each of the points raised, marked using yellow color in the Revised Manuscript with Track Changes.

- Please note that PLOS ONE has specific guidelines on code sharing for submissions in which author-generated code underpins the findings in the manuscript. In these cases, we expect all author-generated code to be made available without restrictions upon publication of the work. Please review our guidelines at https://journals.plos.org/plosone/s/materials-and-software-sharing#loc-sharing-code and ensure that your code is shared in a way that follows best practice and facilitates reproducibility and reuse.

**Response:** We confirm that all author-generated code has been made publicly available on GitHub in accordance with PLOS ONE’s code sharing policy:
 [https://github.com/AliAbbasAbbod/YOLOv10-Arson-Optimization](https://github.com/AliAbbasAbbod/YOLOv10-Arson-Optimization)

- We suggest you thoroughly copyedit your manuscript for language usage, spelling, and grammar. If you do not know anyone who can help you do this, you may wish to consider employing a professional scientific editing service.

**Response:** The manuscript has been carefully proofread for grammar and clarity. All suggested language issues have been addressed and corrected in the revised version.

- We note that your Data Availability Statement is currently as follows: [All relevant data are within the manuscript and its Supporting Information files.

**Response:** We confirm that the minimal dataset required to replicate our results has been made publicly available via the following Kaggle repository:
 <https://www.kaggle.com/datasets/aliabbasabbod/arson-dataset>
Additionally, supporting files including training logs and evaluation metrics are included in the Supplementary Materials.

- **Comment:** One or more of the reviewers has recommended that you cite specific previously published works. Members of the editorial team have determined that the works referenced are not directly related to the submitted manuscript. As such, please note that it is not necessary or expected to cite the works requested by the reviewer.

**Response:** We have considered the references suggested by the reviewers, but only incorporated those that are strongly relevant to the objectives and scope of this study.

- **Comment:** Please include a copy of Table 5 which you refer to in your text on page 23.

**Response:** Thank you for your comment. We have ensured that all tables, including Table 5, are properly included and correctly referenced in the revised manuscript.

**Reviewer 2**

We acknowledge your comments and have taken them into sincere consideration. Below is our response to each of the points raised, marked using yellow color in the revised manuscript with track changes.

- **Comment:** Clarity and English Language Quality: The manuscript contains grammatical and syntactic issues throughout. Many sentences are long, redundant, or awkwardly phrased. A professional English language editing service is highly recommended. Example: “Besides, damage early minimizes loss of lives, obedience to authorities…” → This is unclear. Likely intended to be something like: “Early damage detection minimizes loss of life and ensures compliance with safety regulations.”.

**Response:** We have carefully proofread and revised the entire manuscript, including the abstract, methodology, results, and other sections, to enhance grammar, syntax, and readability.

***Revised part:*** *Early detection of damages reduces loss of life, compliance with authorities and preservation of the environment.*

- **Comment:** The choice of hyperparameters (e.g., lr0, lrf, mo, wd) should be better justified based on domain knowledge or ablation studies.

**Response:** The hyperparameters (lr0, lrf, mo, and wd) were selected due to their well-documented and significant impact on convergence speed, generalization, and overfitting control, as highlighted in recent deep learning literature. In the revised manuscript (Section 2.3, Table 2.3), we explicitly justified their importance and provided their recommended ranges based on previous studies. Moreover, these parameters were prioritized because of their critical role in real-time object detection tasks, such as arson detection, where rapid response and high precision are essential.

***Revised part:*** In modern ML and DL sets, hyperparameters are important in determining model performance. Hyperparameters such as learning rate (lr0), Learning Rate Factor (lrf), Momentum (mo) and weight decay (wd) enhance quick convergence beside ensuring good generalization capacity that greatly reduces other risks such as overfitting or underfitting (1). Balancing these parameters is most important for a task when a certain set of parameters must exceed certain values; for example, for object detection in conditions of active movement arson detection. A rapid response is essential in such cases, making precision a top priority. The primary difficulties regarding hyperparameter tuning have been solved by growing more sophisticated approaches which draw on the natural and evolutional procedures. These optimizations utilize various approaches in the appropriate searching for solutions within a solution space efficiently, with an aim of selecting hyperparameters that will enhance model performance while at the same time reducing the costs of computation. These hyperparameters were carefully chosen according to the literature on DL, including their significance and possible influence (2). A description and the recommended ranges of key YOLO hyperparameters are presented in Table ‎1.

Table 1: Description and Recommended Ranges of Key YOLO Hyperparameters

| Hyperparameter Name & Reference | Description | Recommended Range |
| --- | --- | --- |
| lr0 (3, 4) | controls how quickly the model modifies its weights while it is being trained. A greater lr may speed up the model's convergence, but it may also cause the model to miss the ideal weights, which would impair performance. Conversely, a lower (lr) might result in more steady convergence but could also lengthen the time it takes the model to find the optimal solution | [0-1] |
| lrf (5) | is usually used to adjust the (lr) during the training period. This factor allows the (lr) to be changed, helping to achieve a better balance during the learning process and avoid the problem of gradient descent | [0-1] |
| mo (6) | is a technique used to speed up the training process in the right direction and reduce oscillation | [0-1] |
| wd (7) | is a regularization method that targets the size of the model's weights by applying a penalty to the loss function. The weight decay parameter regulates the severity of this penalty. This method can improve the model's performance on new data and prevent overfitting | [0-1] |

- **Comment:** The rationale behind specific values (e.g., population size = 20, epochs = 20) should be clarified—were these empirically determined?

**Response:** The values for epochs (20) and population size in the optimization algorithms (20 individuals) were empirically determined through preliminary trials to achieve a balance between numerical convergence, computational efficiency, and acceptable performance. Furthermore, these fixed values were applied consistently across all experiments to ensure fair and reliable comparative evaluations. This clarification has been explicitly added to the revised manuscript.

***Revised part:*** *In all experiments, the epochs were 20 and the population size used in optimization algorithms was 20 individuals. The values that were empirically studied in this paper were chosen to provide both numerical convergence and acceptable performance, and maintain computational efficiency. Fixing them also ensured consistent and fair comparative evaluations.*

- **Comment:** While the hybrid GWO-BBOA shows better results than individual algorithms, it’s unclear how statistically significant these differences are. A statistical significance test (e.g., t-test or ANOVA) should be included.

**Response:** We have addressed this concern by performing statistical significance tests to validate the performance differences. Specifically, we conducted five independent runs for each algorithm and applied pairwise T-tests with Bonferroni correction across all major evaluation metrics. The results have been added to the revised manuscript in Section 3.3.5 (Statistical Significance Analysis) and presented in Table 6**.**

**Revised part**:

Statistical Significance Analysis

In order to determine the statistical significance of the performance improvement obtained by the proposed Hybrid GWO-BBOA over individual optimization algorithms (PSO, GWO, and BBOA), five independent runs were performed of each algorithm. To compare the performance of the algorithms in all the major evaluation metrics, Precision, Recall, mAP@0.50, and mAP@0.50:0.95, the pairwise T-tests with Bonferroni correction were used. The findings, summarized in Tables 6, indicate that the proposed GWO-BBOA is considerably better than PSO and BBOA in most metrics with highly significant differences in Precision, Recall and mAP@0.50. Conversely, the performance gap between GWO and GWO-BBOA is usually minor and insignificant in certain measures, including mAP@0.50 and mAP@0.50:0.95.

**Table 6. Pairwise T-test results** **Comparing the Proposed GWO-BBOA with PSO, GWO, and BBOA**

| **Metric** | **Algorithm** | **T-value** | **p-value** | **Statistical Significance** |
| --- | --- | --- | --- | --- |
| Precision | PSO | -6.10 | 0.001 | Highly Significant |
|  | GWO | -2.12 | 0.078 | Not Significant |
|  | BBOA | -7.35 | <0.001 | Highly Significant |
| Recall | PSO | -5.48 | 0.002 | Highly Significant |
|  | GWO | -3.34 | 0.024 | Significant |
|  | BBOA | -2.98 | 0.033 | Significant |
| mAP@0.50 | PSO | -6.15 | 0.001 | Highly Significant |
|  | GWO | -1.20 | 0.286 | Not Significant |
|  | BBOA | -3.52 | 0.019 | Significant |
| mAP@0.50:0.95 | PSO | -4.65 | 0.006 | Significant |
|  | GWO | 0.41 | 0.699 | Not Significant |
|  | BBOA | -0.92 | 0.397 | Not Significant |

On the whole, these results prove that the hybrid optimization method is consistently better than PSO and BBOA and that it can perform as well as GWO in some settings. This shows the usefulness of hybrid optimization methods in improving the performance of deep learning models in detecting arson.

- **Comment:** The comparison with Singh et al. (2020) is weak, as the models, datasets, and objectives differ greatly. Instead, a comparison with similar object detection or fire/arson detection papers using YOLO variants would strengthen the discussion.

**Response:** We agree that the comparison with Singh et al. (2020) presents limitations due to the differences in model architecture, dataset type, and application focus. In response, we have strengthened the comparison section by incorporating a recent and more relevant study by Abbod et al. (2025), which utilized YOLOv9-s for arson event detection using object-based image frames. This addition provides a more direct and meaningful benchmark against our proposed YOLOv10 model with GWO-BBOA optimization. The updated comparison highlights the performance improvements achieved through advanced hyperparameter tuning techniques, especially in real-time fire detection contexts.

**Revised part**:

Comparison with Previous Studies

In order to test the capability of the proposed GWO-BBOA hybrid optimization approach for hyperparameter tuning in the YOLOv10 deep learning model for anomaly detection, it is compared with the works in the literature, which used other deep learning models for anomaly detection. While direct comparisons are challenging due to differences in datasets, models, and evaluation metrics, a relative performance analysis provides valuable insights into the advantages of our approach.

Table 7 presents a comparison between our study, Abbod et al. and Singh et al.

| Criteria | Our Study | Abbod et al. | Singh et al. |
| --- | --- | --- | --- |
| Model | YOLOv10s + Optimization | YOLOv9-s | InceptionV3 + RNN |
| Optimization Techniques | PSO, GWO, BBOA, GWO-BBOA | Default  hyperparameter tuning | Manual hyperparameter tuning |
| Application | Arson detection | Arson detection | General anomaly detection in surveillance videos |
| Data Type | Image frames (object-based detection) | Image frames from videos | Video sequences (temporal anomaly detection) |
| Dataset Size | 2,182 images from 53 videos | 2,182 images from 53 videos | Not specified |
| Best Performance | Precision = 0.750, Recall = 0.620, mAP@0.50 = 0.705 | Precision = 0.445,  Recall = 0.507,  mAP@0.50 = 0.456 | Precision, recall, and mAP@0.50 are not mentioned but an overall improvement Overall anomaly classification |
| Limitations | Focuses only on arson detection | Limited recall and precision; moderate performance under confidence tradeoff | No optimization of hyperparameters |

One of the most applicable studies in the recent past is that by Abbod et al. (2025) that used YOLOv9-s to detect arson events on surveillance video frames as shown in Table **7**. Although their method proved that the YOLO-based models can be used in this area, they did not optimize default parameters. On a dataset of 2,182 images extracted in 53 videos, their model reached a precision of 0.445, recall of 0.507 and mAP@0.50 of 0.456. By contrast, we used GWO-BBOA metaheuristic tuning with YOLOv10 and achieved much better performance (precision = 0.750, recall = 0.620, mAP@0.50 = 0.705), which shows the obvious benefit of using more advanced methods of hyperparameter tuning.

While Singh et al. (2020), used the InceptionV3 + RNN model which is used to predict video sequences over time, our model focuses on real-time object detection within individual frames. Our interpretation of this distinction suggests that even though YOLOv10 is a very efficient tool in the task of detecting arson in still images, a sequential model, such as RNN, may be more effective in making inferences based on patterns at different frames in long term anomaly detection. The variation in approach to this indicates the necessity of establishing such models in accordance with the particular application demands, as real-time detection prospers from the object-based models like YOLO, while temporal models like RNN might be utilized for event-based anomaly detection.

These comparative findings open the door to a future study of hybrid models and general anomaly detection tasks, which are discussed in the following sections.

- **Comment:** Lack of Visual Results: There is a notable absence of qualitative visual results (e.g., bounding box detection outputs for arson scenes). Including detection outputs for different scenarios (e.g., low light, occlusions) would demonstrate practical robustness.

**Response:** We agree that including qualitative visual results across challenging scenarios is essential to demonstrate the robustness of our arson‑detection pipeline. To address this, we have now added new Figures 9 showing Grad‑CAM heatmaps.

**Revised part**:

3.3.7	Grad-CAM Heatmaps for Arson Detection

To assess the interpretability and effectiveness of the model, visual explanation techniques such as heatmaps are employed. These tools help clarify and validate the model’s predictions by showing which areas of the image the model is focused on while making predictions. A heatmap transforms complex data into a vibrant, color-coded matrix (40). As shown in Figure ‎8A, the original image is presented without any heatmap. In Figure 8B, we show bounding‑box—tightly enclosing both the person and the jug—to indicate the model’s detected region of interest. Figure ‎ 8C, illustrates the heatmap generated by the YOLO v10 model, where attention is more concentrated on specific regions of the image, particularly the area around the fire. In the context of arson detection, the YOLO v10 model’s decision-making process can be analyzed by interpreting its attention through these heatmaps. As fire detection requires distinguishing between accidental fires and deliberate fires (arson), the heatmaps serve to show which features such as human behavior, fire spread patterns, or unusual objects near the fire are deemed most relevant by the model. The red and yellow regions in the heatmap correlate with the features that the model associates with arson, confirming that it is focusing on the critical elements of the image that indicate intentional fire-setting behavior. Visualization tools like heatmaps allow researchers and practitioners to understand the underlying reasons for a model’s decisions, increasing confidence in its ability to detect arson and preventing FPs. However, with these interpretability techniques, we can assess the model’s performance in different scenarios. Depending on the situation the model should concentrate more on human interactions and behaviors and less on suspicious activities in case of purposeful fire and vice versa in case of an accidental fire. This flexibility in the model’s interpretability is crucial for ensuring accurate and reliable arson detection in real-world environments.

Fig 9. Visualized results for a single arson scene, showing (A) the original image, (B) YOLO v10 bounding‑box, and (C) Grad‑CAM heatmap overlay.

Finally, the experimental evaluation of the YOLO v10 model showed that it is capable of performing effective arson detection when appropriately sized and using advanced hyperparameter tuning techniques.

- **Comment:** Dataset Limitations and Augmentation: Although augmentation expands the dataset, the original number of 290 frames from 53 videos is still quite small for training deep models. This limitation needs to be acknowledged and discussed more thoroughly, especially its effect on model generalization.

**Response**: The dataset limitation and its effect on model generalization have been explicitly acknowledged in the revised manuscript. A note was added in the data preparation section, and future work now includes plans to expand the dataset and explore synthetic data generation (e.g., GANs) to enhance generalization**.**

**Revised part**:

**Data Preparation and Dataset Exploration**

(……… While we use data augmentation techniques to make the dataset appear larger, they are not a complete replacement for diverse, real-world samples. Therefore, the model's capacity to generalize to new arson scenarios and environmental conditions may be hampered by the lack of truly independent frames.

**Conclusions and Suggestions for Future Work**

obtaining a far more extensive and diverse collection of fire video footage, ideally encompassing a range of indoor and outdoor environments, camera perspectives, and sources of ignition. Simultaneously, we advise investigating sophisticated synthetic data generation methods, like generative adversarial networks (GANs), to produce lifelike fire simulations that enhance actual video. To improve model generalization and guarantee reliable performance in arbitrary surveillance scenarios, such efforts will be essential.

- **Comment:** Real-World Deployment Discussion: The conclusion briefly mentions integration with real-time systems. However, more elaboration is needed: What are the latency constraints? Can the model run on edge devices (e.g., security cameras)? What challenges are foreseen in such deployments?

**Response:** A paragraph was added to the conclusion section to elaborate on the real-world deployment challenges, including latency constraints, edge device compatibility, and practical limitations such as memory and energy requirements. This addition provides a clearer view of how the proposed YOLOv10s model could function in live surveillance environments.

**Revised part**:

Although the model has demonstrated strong performance in controlled environments, deploying YOLOv10 with the GWO-BBOA optimized parameters in real-world systems introduces practical challenges. One major concern is latency, especially in time-sensitive scenarios such as fire or arson detection. The model must deliver inference within milliseconds to be viable for live surveillance systems. YOLOv10s, being a lightweight variant, offers promising latency levels (sub-100ms on modern GPUs), but edge deployment requires further compression and quantization. Moreover, deploying the model on edge devices like security cameras or IoT systems brings resource constraints such as limited memory, processing power, and battery life. While YOLOv10s is portable, real-time performance on edge may require pruning or using accelerators like NVIDIA Jetson or Google Coral.

Minor Comments and Technical Corrections:

- **Comment:** Abstract: Avoid listing too many metrics in the abstract unless absolutely necessary.

**Response:** We revised the abstract to include only one key metric (recall = 0.620) to highlight the model’s effectiveness without overwhelming the reader with multiple numerical values**.**

**Revised part**:

Experimental results show that the proposed GWO-BBOA approach outperforms traditional optimization methods, achieving a recall of 0.620. This indicates its enhanced ability to detect true arson events.

- **Comment:** Introduction: The statement "Accidental fires can also cause disastrous effects such causing fires on purpose" is unclear and contradictory.

**Response:** We revised the sentence to remove the contradiction and clarify the focus on arson as a deliberate and harmful act.

**Revised part:** Among these abnormalities, is arson, which is predicted to threaten some societies because it causes property damage and loss of life

- **Comment:** Section 2.3.4: The pseudocode is helpful but could benefit from line numbers and a figure/table format for clarity.
- **Response:** The pseudocode has been revised and reformatted as a labeled figure with hierarchical line numbers for improved clarity and readability (see Figure 6 in Section 2.3.4).
- **Comment:** References: Make sure the reference style is consistent. Some citations are numbered inline; others are formatted inconsistently.

**Response:** We have carefully revised all in-text citations to ensure consistency with the numerical referencing style. All references are now formatted uniformly.

**Reviewer 3**

We acknowledge your comments and have taken them into sincere consideration. Below is our response to each of the points raised, marked using yellow color in the revised manuscript with track changes.

**Comment:** While results are convincing, cross-validation or testing on a secondary dataset would enhance the generalizability of the claims. Including confidence intervals or statistical significance tests could further strengthen the validity of the comparisons. Single-run evaluation is implied (no mention of repeated trials or random seeds), which can limit the reproducibility and reliability of the reported performance.
**Response:** To address the concern regarding the reliability and generalizability of the reported performance, we have extended the experimental setup by conducting five independent runs for each optimization algorithm (PSO, GWO, BBOA, and the proposed GWO-BBOA). These repeated trials were executed with different random seeds to ensure reproducibility and minimize the effect of random variation in training outcomes. To further support the statistical validity of the performance comparisons, we included a pairwise T-test with Bonferroni correction across the four major evaluation metrics: Precision, Recall, mAP@0.50, and mAP@0.50:0.95. The results of these tests, now presented in the revised manuscript (Table 5), clearly demonstrate that the performance improvements of the proposed hybrid GWO-BBOA method over PSO and BBOA are statistically significant. Comparisons with GWO showed smaller and in some cases statistically insignificant differences, which are also acknowledged and discussed.While full cross-validation and testing on a secondary dataset were not applied in this study due to computational limitations and data availability, we have now explicitly mentioned this as a limitation in the revised manuscript and proposed it as future work. We appreciate your suggestion, which contributed to a stronger validation framework in the paper.

**Revised part:**

Statistical Significance Analysis

In order to determine the statistical significance of the performance improvement obtained by the proposed Hybrid GWO-BBOA over individual optimization algorithms (PSO, GWO, and BBOA), five independent runs were performed of each algorithm. To compare the performance of the algorithms in all the major evaluation metrics, Precision, Recall, mAP@0.50, and mAP@0.50:0.95, the pairwise T-tests with Bonferroni correction were used. The findings, summarized in Tables 6, indicate that the proposed GWO-BBOA is considerably better than PSO and BBOA in most metrics with highly significant differences in Precision, Recall and mAP@0.50. Conversely, the performance gap between GWO and GWO-BBOA is usually minor and insignificant in certain measures, including mAP@0.50 and mAP@0.50:0.95.

**Table 6. Pairwise T-test results** **Comparing the Proposed GWO-BBOA with PSO, GWO, and BBOA**

| **Metric** | **Algorithm** | **T-value** | **p-value** | **Statistical Significance** |
| --- | --- | --- | --- | --- |
| Precision | PSO | -6.10 | 0.001 | Highly Significant |
|  | GWO | -2.12 | 0.078 | Not Significant |
|  | BBOA | -7.35 | <0.001 | Highly Significant |
| Recall | PSO | -5.48 | 0.002 | Highly Significant |
|  | GWO | -3.34 | 0.024 | Significant |
|  | BBOA | -2.98 | 0.033 | Significant |
| mAP@0.50 | PSO | -6.15 | 0.001 | Highly Significant |
|  | GWO | -1.20 | 0.286 | Not Significant |
|  | BBOA | -3.52 | 0.019 | Significant |
| mAP@0.50:0.95 | PSO | -4.65 | 0.006 | Significant |
|  | GWO | 0.41 | 0.699 | Not Significant |
|  | BBOA | -0.92 | 0.397 | Not Significant |

On the whole, these results prove that the hybrid optimization method is consistently better than PSO and BBOA and that it can perform as well as GWO in some settings. This shows the usefulness of hybrid optimization methods in improving the performance of deep learning models in detecting arson.

**Reviewer 4**

We acknowledge your comments and have taken them into sincere consideration. Below is our response to each of the points raised marked using yellow color in the revised manuscript with track changes.

**Comment: Major Concerns and Suggestions for Improvement:**

1. Lack of Related Work Section: The manuscript does not include a dedicated Related Work section. A detailed comparison with prior work—especially in the areas of fire/arson detection and other optimization methods—is crucial for positioning the novelty and contribution of this work.

**Response:** We agree that including a dedicated Related Work section is crucial for clearly identifying the novelty and positioning of our study. In response, we have revised the manuscript to add a separate Previous Studies section. In this section, we have elaborated on recent and relevant research related to arson detection as well as various optimization algorithms used in deep learning contexts.

**Revised part:**

**Previous Studies**

Previous studies have shown the huge benefit of anomaly detection in improving the accuracy of various deep learning models as well as optimization algorithms, For example, (8) presented rapid determination of whether an unexpected action is odd or suspicious. It is necessary to indicate which frames and segments of the recording feature the unusual activity. Thus, they used deep learning techniques to automate the threat recognition system to minimize the waste of labor and time. It aims to automatically distinguish abnormalities from typical patterns by recognizing aggressive and violent indicators in real time. They plan to apply two distinct deep learning models (CNN and RNN) to detect and categorize high movement levels in the frame. Subsequently, they were able to detect warnings in the event of a threat, highlighting any suspicious activity at that particular moment. In the context of optimization algorithms, (9) considered the use of PSO for DL models using activation functions and batch size. Another advantage of the PSO approach is the ability of swarm-based strategy for searching for the solution space with a reduced chance of convergence to local optima, hence enhancing model accuracy. In contrast, (10) developed GWO (Grey Wolf Optimizer) based on the leadership and hunting mechanisms of real-life grey wolves. The proposed GWO approach offers a positive example of exploring and exploiting experience as demonstrated by the alpha, beta, and delta wolves, which also demonstrates its ability to optimize hyperparameters to address complex problems, including classification and regression. On the other hand, (11) highlighted the idea of BBOA, which is a new approach for dynamic optimization in learning phases and adjusts learning rates for model stability applications like classification and segmentation. Altogether, all these algorithms show special versatility in enhancing the performance, speed, and flexibility of DL frameworks.

The use of a hybrid of multiple optimization algorithms in hyperparameter optimization has been a success in enhancing the overall performance of the model. Furthermore, (12) proposed the integration of GA and XGBoost in fraud detection tasks, with good gains in terms of efficiency and accuracy. This approach highlighted the advantage of combining metaheuristic approaches in handling constraints that may be inherent in them when used singularly.

Finally, (13) evaluated the performance of the YOLOv9 model for detecting arson incidents in surveillance videos. The study demonstrated that while the model achieved a precision of 0.552 overall in arson classification, it could reach a confidence level of 0.933 when predicting certain events. These findings confirm the feasibility of using YOLO-based architectures for arson detection, although further enhancements in model tuning and environmental robustness were suggested for broader applicability. This study forms a baseline for the current work, which aims to improve detection accuracy using the YOLOv10 model with advanced hybrid optimization.

These studies highlight the increasing relevance of integrated metaheuristic approaches for improving the predictive outcomes. Nevertheless, the application of GWO and BBOA in optimizing the state-of-art models such as YOLOv10 has little attention in the literature, such research will fill the gap in the existing knowledge and contribute significantly to the advancements in the field.

2. Insufficient Discussion of Novelty and Challenges: The introduction briefly states the use of YOLOv10 and the hybrid optimization method, but it does not clearly articulate the technical challenges being addressed that prior works have not solved. Please elaborate on:

a) What specific limitations in previous models or optimization strategies this work aims to overcome?

b) Why GWO-BBOA is particularly suitable in this context?

**Response:** In response, we have revised the Introduction and Section 3.3.4 (Hybrid GWO-BBOA) to clearly articulate both the technical challenges addressed and the novelty of our approach.

a) In the Introduction, we now highlight the limitations of prior models and optimization strategies. Specifically, we discuss how traditional techniques such as PSO and GA often suffer from premature convergence and poor performance in noisy surveillance conditions. We also note that YOLOv10, despite its strengths, can fail to detect small-scale or ambiguous fire events in low-light or occluded settings due to suboptimal hyperparameters.

b) In Section 3.3.4, we explain why the proposed hybrid GWO-BBOA method is well-suited to this context. GWO provides structured local exploitation through its hierarchical leadership mechanism, while BBOA contributes adaptive global exploration. Their integration ensures robust, diverse, and well-tuned hyperparameters that improve YOLOv10's detection capability in dynamic environments.

3. No Visualization of Detection Results: To assess the practical effectiveness of the proposed method, the paper should include visual examples of detection results (e.g., bounding boxes overlaid on frames with fire/arson events). This helps reviewers and readers evaluate qualitative performance.

**Response:** In response to your suggestion, we have now included a visual comparison of detection results in the revised manuscript (see Figure 8 in Section 3.3.1). This figure illustrates both the ground truth annotations and the predicted bounding boxes produced by our proposed YOLOv10 model optimized with the hybrid GWO-BBOA algorithm.

4. Lack of Model Input/Output Description: The paper does not clearly explain what kind of data is used as input to the model (e.g., image resolution, frame rate, RGB vs. thermal, etc.) and what format the model outputs. Please provide a clearer overview of the pipeline, including preprocessing (if any) and output interpretation.

**Response:** In response, we have revised Section 3.1 (Data Preparation and Dataset Exploration) to include a detailed description of the model's input and output pipeline. Specifically, we now clarify that the model takes in RGB image frames resized to 640×640 pixels, normalized to the range [0,1], and processed at 25 frames per second. The output is described in terms of bounding box predictions including class labels and confidence scores, and we also highlight YOLOv10’s built-in Dual Label Assignment, which removes the need for external Non-Maximum Suppression (NMS).

5. No Code Availability: To ensure reproducibility and to support future research, the authors should provide a public link to their code. This is particularly important for works involving novel optimization strategies applied to established models.

**Response:** We have addressed this by uploading all relevant code and configurations to a public GitHub repository to ensure reproducibility and support future research. The repository is available at the following link:

<https://github.com/AliAbbasAbbod/YOLOv10-Arson-Optimization>

This includes the implementation of all four optimization algorithms, training scripts, and setup instructions.

**Reviewer 5**

## We acknowledge your comments and have taken them into sincere consideration. Below is our response to each of the points raised marked using yellow color in the revised manuscript with track changes.

- **Comment:** The reproduction of Figure 2 from the original YOLOv10 paper is not presented clearly. All four figures are in low resolution, which affects readability. It is strongly recommended to use high-resolution or vector graphics when applicable.

**Response:** Thank you very much for your valuable comment regarding Figure 2. Your feedback has been carefully considered. Some of the subfigures have been replaced with higher-resolution versions to enhance clarity, while others have been improved using appropriate image processing tools. Every effort has been made to ensure that all components are now clearer and more readable, while preserving the scientific accuracy and original content. I hope the updated version meets the expected quality standards.

- **Comment:** Baselines are essential for evaluation. Please include the training results of YOLOv10 using the original hyperparameters in Table 3 for comparison.

**Response**: We fully agree that including baseline results is critical for evaluating the effectiveness of the proposed optimization method. Accordingly, we have added the performance of the YOLOv10 model trained with default hyperparameters (i.e., without any optimization) to Table 4 as the Baseline. This configuration achieved a precision of 0.727, recall of 0.470, and mAP@0.50 of 0.558, serving as a reference point. We have also revised the corresponding paragraph to highlight that the proposed GWO-BBOA optimization achieved significant improvements over the baseline, particularly in recall and mAP.

- **Comment:** It seems that two pictures augmented from the same original picture were split across the training set and test sets. Arson002_x264_frame_0060.jpg from the test set and Arson002_x264_frame_0059_aug_out_aug_1.png from the training set are very similar. To prevent data leakage and ensure the validity of results, such overlap should be avoided.

**Response:** We would like to clarify that the original dataset (290 images) was first split into training (70%), validation (20%), and test (10%) sets before applying any augmentation. Data augmentation was then performed separately within each subset, ensuring that no augmented version of a training image appears in the test set. Additionally, although the reviewer referenced two images (frame 0059 and 0060) that appear similar, these are distinct frames from the original video sequence (i.e., neighboring but not identical frames). We acknowledge the visual similarity due to the temporal nature of video data, but they originate from different source frames, and thus no data leakage occurred during training or evaluation.

.

- **Comment:** In line 339, it’s unclear whether 100 epochs were applied to all training. Since different hyperparameters may lead to the model convergence at different epochs. It’s advisable to report the stopping strategy used in this research. Reporting metrics such as mAP or loss at each epoch would support the experimental results.

**Response:** Based on the results presented in Table 2, 100 epochs provided the best trade-off between performance metrics (such as precision and recall) and training time. Accordingly, we adopted a fixed value of 100 training epochs for all subsequent experiments, including those involving hyperparameter variations.

No early stopping strategy was applied in this study. The decision to use a fixed number of epochs was made to ensure consistent training conditions and fair comparison across different experimental settings. Furthermore, in response to your suggestion, we have added a detailed plot (Figure 7) illustrating the evolution of key training losses and evaluation metrics (including mAP@0.5 and mAP@0.5:0.95) over the course of 100 epochs. This plot confirms stable convergence and supports the selected training configuration.

We hope these additions sufficiently address your concerns and enhance the clarity and completeness of the experimental setup.

.

- **Comment:** Regarding Table 4, more relevant and recent works may serve as better points of comparison. Suggested alternatives include: https://doi.org/10.1016/j.eswa.2023.119741 and <https://doi.org/10.3390/app14135841>.

**Response:** Thank you for your valuable comment and for suggesting recent and relevant studies. We have carefully reviewed the two articles:

Özcan et al. (2024) [https://doi.org/10.3390/app14135841], which investigates the improvement of YOLO-based object detection in adverse weather conditions using metaheuristic algorithms.

Karaman et al. (2023) [https://doi.org/10.1016/j.eswa.2023.119741], which explores the use of the Artificial Bee Colony (ABC) algorithm to optimize activation functions and hyperparameters for YOLOv5 in real-time polyp detection.

Both studies are highly relevant in the context of applying metaheuristic optimization to improve YOLO-based object detection performance. However, they focus on different application domains and datasets (autonomous driving in AWCs and medical imaging, respectively). While their methodologies provide meaningful insights, their direct comparability to our work is somewhat limited due to differences in dataset characteristics and application focus.

In response to your suggestion, we have updated Table 4 and the related discussion to include a directly related and more comparable work that uses the same dataset as ours. This inclusion allows for a fairer and more informative comparison, as it evaluates methods under the same experimental conditions.

We appreciate your recommendation, which helped improve the contextual relevance and comprehensiveness of our comparative analysis.

- **Comment:** The program mentioned on line 138 needs to be properly cited.

**Response:** The citation for the LabelImg program has been added as reference (20).

- **Comment:** Grammatical correctness is needed at lines 35, 149, and 333.

**Response:** We have carefully proofread and revised the entire manuscript, including the abstract, methodology, results, and other sections, to enhance grammar, syntax, and readability.

**Revised part:**

35- Among these abnormalities, is arson, which is predicted to threaten some societies because it causes property damage and loss of life

149- The "one-to-many'' technique provides hints and suggestions while the "one-to-one" strategy eliminates the need for NMS in inference procedures to improve efficiency.

333- For the purpose of training dataset experiments, 100 and 150 epochs were performed to determine the optimal number of epochs for training the model.

- **Comment:** A range should be given at each line of 389, 390, and 392.

**Response:** The values mentioned in lines 389, 390, and 392 are fixed parameters rather than variable ranges. Therefore, we have clarified this in the revised manuscript by explicitly stating these fixed values in the text.

**Revised part:** In all experiments, the epochs were fixed at 20, and the population size used in optimization algorithms was 20 individuals to ensure consistent comparative evaluations. Moreover, YOLOv10 was trained using the AdamW optimizer with a fixed batch size of 96.
