# Supplementary material for: Optimizing hyperparameters of YOLOv10 for arson detection using advanced optimization algorithms
Source: PLoS One. 2025 Oct 7;20(10):e0333374. doi: 10.1371/journal.pone.0333374 (PMC12503287; doi:10.1371/journal.pone.0333374)
Supplement: Attachment — Submitted filename: Response_to_Reviewers_auresp_2.docx [file pone.0333374.s003.docx]

***Response to Reviewers***

**Manuscript Title:** Optimizing Hyperparameters of YOLOv10 for Arson Detection Using Advanced Optimization Algorithms

**Manuscript Number:** PONE-D- 25-07942

We would like to express our sincere appreciation to the editor and the reviewers for their insightful comments and valuable suggestions, which have greatly contributed to enhancing the clarity and overall quality of our manuscript. We have carefully addressed all concerns and implemented the recommended changes accordingly. The following sections present our detailed responses and highlight the modifications made based on the reviewers' feedback. We are grateful for your constructive input and the opportunity to improve our work through this review process.

**#Academic Editor**

We acknowledge your comments and have taken them into sincere consideration. Below is our response to each of the points raised, marked using yellow color in the Revised Manuscript with Track Changes.

Journal Requirements:

- If the reviewer comments include a recommendation to cite specific previously published works, please review and evaluate these publications to determine whether they are relevant and should be cited. There is no requirement to cite these works unless the editor has indicated otherwise.
- Please review your reference list to ensure that it is complete and correct. If you have cited papers that have been retracted, please include the rationale for doing so in the manuscript text, or remove these references and replace them with relevant current references. Any changes to the reference list should be mentioned in the rebuttal letter that accompanies your revised manuscript. If you need to cite a retracted article, indicate the article’s retracted status in the References list and also include a citation and full reference for the retraction notice.

  **Response:** We have carefully reviewed the journal’s requirements regarding references. No retracted papers were cited, and all references are complete and accurate. Therefore, no changes were necessary in the references section of the revised manuscript.

Reviewer #3: Met the minimum standards of the journal.
Almost addressed the issues raised by the reviewers.
Revision version is much better than the original one.

**Response:** We sincerely thank the reviewer for the positive feedback and for acknowledging the improvements made in the revised version.

Reviewer #4: I appreciate authors' feedback. All my concerns are addressed. Therefore, I would vote for acceptance.

**Response:** We are grateful to the reviewer for the constructive evaluation and are pleased that all concerns were addressed satisfactorily.

**Reviewer #5**

## We acknowledge your comments and have taken them into sincere consideration. Below is our response to each of the points raised marked using yellow color in the revised manuscript with track changes.

- **Comment:** The authors addressed all of my comments, and the updated version shows improvement. However, some low-level mistakes remain evident, which raises concerns that the authors may have rushed to complete the revision.All figures are still in low resolution. Some characters in Figures 1 and 5 are unreadable. I recommend that the authors double-check these. Line 385: “Fig 1. The Flowchart of the Hybrid GWO-BBOA Optimization Process.” This should refer to Figure 5, not Figure 1. The sentence: “We have added the performance of the YOLOv10 model trained with default hyperparameters (i.e., without any optimization) to Table 4 as the Baseline.” I believe the authors meant Table 5, not Table 4.

**Response:**

We sincerely thank the reviewer for the careful evaluation and constructive feedback. We have carefully revised the manuscript to address all the points raised:

- Figure quality (Figures 1 and 5):

All figures have been regenerated at high resolution (minimum 300 dpi) with improved readability of characters, labels, and flowchart text. The updated figures have also been checked using the PACE tool recommended by the journal to ensure compliance with PLOS ONE standards.

- Incorrect figure reference (Line 385):

The incorrect reference has been corrected. The sentence now properly refers to Figure 5 instead of Figure 1.

- Incorrect table reference (Baseline results):

The sentence has been corrected to refer to Table 5 instead of Table 4, which now clearly contains the baseline YOLOv10 (default hyperparameters) performance results.

All the above corrections are highlighted in the revised manuscript (track changes) for easy verification.
